# Supplementary material for: The global human impact on biodiversity
Source: Nature. 2025 Mar 26;641(8062):395–400. doi: 10.1038/s41586-025-08752-2 (PMC12058524; doi:10.1038/s41586-025-08752-2)
Supplement: Supplementary file 1 — Supplementary Figs. 1–7, Table 1 and references. [file 41586_2025_8752_MOESM1_ESM.pdf]

nature portfolio

https://doi.org/10.1038/s41586-025-08752-2

Supplementary information

The global human impact on biodiversity

In the format provided by the authors and unedited

Supplementary Information for:

The global human impact on biodiversity

François Keck*[,1,2], Tianna Peller[1,2], Roman Alther[1,2], Cécilia Barouillet[3,4], Rosetta Blackman[1,2], Éric Capo[5], Teofana Chonova[6], Marjorie Couton[1,2], Lena Fehlinger[7], Dominik Kirschner[2,8,9], Mara Knüsel[1,2], Lucile Muneret[10,11], Rebecca Oester[1,2,12], Kálmán Tapolczai[13,14], Heng Zhang[1,2], Florian Altermatt*[,1,2]

[1] Department of Evolutionary Biology and Environmental Studies, University of Zurich, Winterthurerstr. 190, CH-8057 Zürich, Switzerland

[2] Eawag, Swiss Federal Institute of Aquatic Science and Technology, Department of Aquatic Ecology, Überlandstrasse 133, CH-8600 Dübendorf, Switzerland

[3] INRAE, Université Savoie Mont Blanc, CARRTEL, 74200, Thonon-les-Bains, France

[4] Pôle R&D ECLA, CARRTEL, 74200, Thonon-les-Bains, France

[5] Department of Ecology and Environmental Science, Umeå University, Linnaeus väg 4-6, 907 36, Umeå, Sweden

[6] Eawag, Swiss Federal Institute of Aquatic Science and Technology, Department Environmental Chemistry, Überlandstrasse 133, CH-8600 Dübendorf, Switzerland

[7] GEA Aquatic Ecology Group, University of Vic - Central University of Catalonia, Carrer de la Laura 13, 08500 Vic, Spain

[8] Department of Environmental Systems Science, Institute of Terrestrial Ecosystems, Ecosystems and landscape evolution, ETH Zürich, CH-8092, Zürich, Switzerland

[9] Department of Landscape Dynamics & Ecology, Swiss Federal Research Institute WSL, Zürcherstrasse 111, 8903 Birmensdorf, Switzerland.

[10] INRAE, Université Paris-Saclay, AgroParisTech, UMR Agronomie, 91120, Palaiseau, France

[11] INRAE, Agroécologie, Institut Agro, Univ. Bourgogne, Univ. Bourgogne Franche-Comté, F-21000, Dijon, France

[12] Institute of Microbiology, University of Applied Sciences and Arts of Southern Switzerland, via Flora Ruchat Roncati 15, CH-6850 Mendrisio, Switzerland

[13] HUN-REN Balaton Limnological Research Institute, Klebelsberg Kuno Street 3, H-8237 Tihany, Hungary

[14] National Laboratory for Water Science and Water Security, HUN-REN Balaton Limnological Research Institute, Klebelsberg Kuno Street 3, H-8237 Tihany, Hungary

* Corresponding authors

Supplementary Information

Supplementary Methods **3**
S1. Meta-analytical framework 3
Figure S1 3
S2. Structured query used on Web of Science 4
Table S1 4
S3. Modified PRISMA flowchart 6
Figure S2 6
S4. Additional information on data collection and extraction 7
Figure S3 8
S5. Impact of additional samples in PCoA/NMDS on effect size estimation 8
Listing S1 9
Figure S4 11
Figure S5 12
S6. Assessment of publication bias 13
Figure S6 14
S7. Spatial distribution of reference and impacted sites 15
Figure S7 16

Supplementary References **17**

Supplementary Methods

S1. Meta-analytical framework

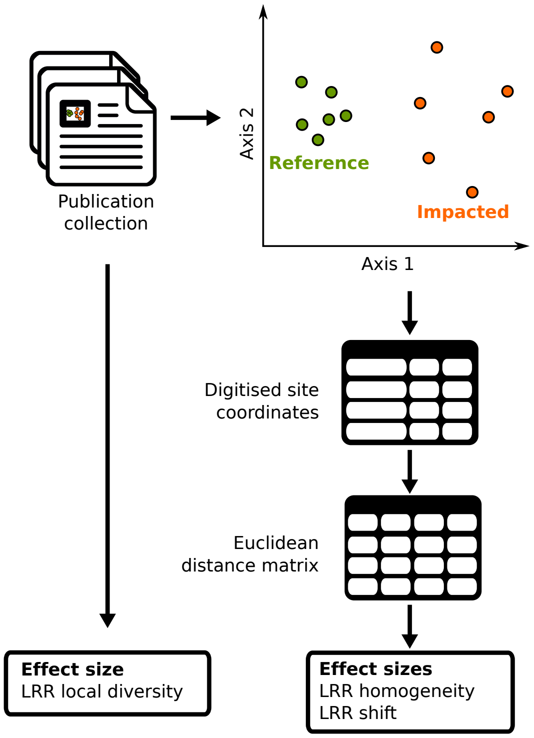

Figure S1 Principal coordinates analysis (PCoA) and non-metric multidimensional scaling (NMDS) are two methods that can be used to display the information contained in a matrix of dissimilarity. Distances between communities on the plot reflect, in two dimensions, their dissimilarities. Therefore, these plots can be used to assess the changes in community structure (shift in point cloud location) and the changes in similarity (shift in point cloud dispersion). The meta-analytic framework starts from a plot of projection of the distances between communities, comparing reference with impacted communities as frequently reported in publications. Community coordinates are manually extracted from the plot using a digitising tool. Coordinates are then translated into a pairwise distance matrix. Finally, these distances are used to compute two measures of effect-size representing the changes in terms of homogeneity and shift in composition.

S2. Structured query used on Web of Science

Facets	Keywords
Publications…	DT=(Article OR Early Access OR Letter OR Note)
…in relevant scientific disciplines…	(WC=(MULTIDISCIPLINARY SCIENCES) OR WC=(Agriculture, Multidisciplinary) OR WC=(Biodiversity Conservation) OR WC=(Ecology) OR WC=(Environmental Sciences) OR WC=(Environmental Studies) OR WC=(Fisheries) OR WC=(Forestry) OR WC=(Limnology) OR WC=(Marine & Freshwater Biology) OR WC=(Microbiology) OR WC=(Oceanography) OR WC=(Plant Sciences) OR WC=(Soil Science) OR WC=(Zoology))
…from 1960…	PY=(1960-2022)
…about community and diversity…	TS=(communit* OR diversit* OR assemblage*)
…in the context of ecology…	TS=(ecolog* OR environment* OR biolog*)
…focusing on beta-diversity…	TS=(beta-diversity OR "beta diversity" OR diversity OR structure OR turnover OR composition OR change OR changes)
…changes…	TS=(reference$ OR baseline$ OR impact* OR effect$ OR pressure$ OR change OR modification$ OR shift$ OR response$)
…caused by human activities.	TS=("pollution$" OR "pollutant$" OR "contamination" OR "eutrophication" OR "municipal waste" OR "hazardous chemicals" OR "hazardous substance$" OR "waste water dumps" OR "wastewater" OR "pesticides" OR "open waste dumps" OR "dump sites" OR "heavy metals" OR "micro-pollutants" OR "waste landfills" OR "organic waste" OR "construction waste" OR "demolition waste" OR "hazardous waste" OR "habitat?change$" OR "habitat?loss" OR "habitat destruction" OR "habitat conversion" OR "habitat modification$" OR "habitat fragmentation" OR "loss of connectivity" OR "forest fragmentation" OR "deforestation" OR "land?use change$" OR "land?use" OR "landscape change" OR "urbani?ation" OR "urban expansion" OR "urban land expansion" OR "agricultural expansion" OR "intensive agriculture" OR "crop lands expansion" OR "grazing lands expansion" OR "infrastructure development" OR "intensified land management systems" OR "tree plantation$" OR "industrial development" OR "agroforestry" OR "human encroachment" OR "managed forest" OR "transformation of natural ecosystems" OR "human use-dominated ecosystems" OR "anthromes" OR "anthropic biomes" OR "road construction" OR "road expansion" OR "road densit*" OR "dam construction" OR "port construction" OR "sea-ice change" OR "seascape change" OR "change$ in seascape" OR "loss of coastal habitats" OR "degradation of coastal habitats" OR "loss of coral reefs" OR "loss of seagrasses" OR "loss of mangroves" OR "loss of salt marshes" OR "fragmentation of seascape" OR "loss of wetlands" OR "conversion of coastal wetlands" OR "loss of inland natural wetlands" OR "changes in sediment flows" OR "reduction in sediment inputs" OR "monoculture plantation$" OR "land degradation" OR "degraded land" OR "soil degradation" OR "surface sealing" OR "soil compaction" OR "soil acidification" OR "soil fertility loss" OR "salinization" OR "soil sealing" OR "soil erosion" OR "soil artificiali?ation" OR "land artificiali?ation" OR "organic matter depletion" OR "rangeland degradation" OR "freshwater degradation" OR "forest degradation" OR "loss of wetlands" OR "loss of hydrological functions" OR "biomass extraction" OR "biomass materials extraction" OR "resource extraction" OR "raw material extraction" OR "domestic extraction" OR "harvested biomass" OR "grazed biomass" OR "animal biomass extraction" OR "plant-based biomass extraction" OR "minerals extraction" OR "gold extraction" OR "sand extraction" OR "gravel extraction" OR "limestone extraction" OR "clays extraction" OR "fossil energy carriers extraction" OR "coal extraction" OR "crude oil extraction" OR "natural gas extraction" OR "shale gas extraction" OR "hydrated gas extraction" OR "shale oil extraction" OR "timber extraction" OR "construction materials extraction" OR "fossil fuels extraction" OR "groundwater extraction" OR "surface water extraction" OR "fuelwood collection" OR "non-timber natural resource extraction" OR "extractive industry" OR "wood extraction" OR "charcoal extraction" OR "ecosystem-derived fuels extraction" OR "fuelwood extraction" OR "over$fishing" OR "over$exploitation" OR "over$grazing" OR "over$hunting" OR "over$harvesting" OR "over$fished" OR "over$exploited" OR "over$hunted" OR "over$grazed" OR "over$harvested" OR "climate change" OR "global warming" OR "climate warming" OR "ocean acidification" OR "glacier retreat" OR "sea$level rise" OR "invasive species" OR "biological invasion" OR "invasive" OR "invasion$" OR "alien species" OR "introduced species" OR "non?native species" OR "fishery" OR "fisheries" OR "aquaculture" OR "industrial fishing" OR "fish stocks" OR "shrimp farming" OR "salmon farming" OR "agriculture" OR "crop production" OR "fertilization" OR "agricultural expansion" OR "cattle" OR "agricultural intensification" OR "livestock" OR "pasture" OR "food crops" OR "grazing lands" OR "agricultural systems" OR "logging" OR "wood fuel harvest" OR "timber" OR "mining" OR "fossil fuel production" OR "shale oil" OR "shale gas" OR "offshore oil" OR "offshore gas" OR "dams" OR "reservoirs" OR "hydropower generation" OR "illegal activities" OR "illegal fishing" OR "illegal forestry" OR "illegal logging" OR "illegal poaching" OR "tourism" OR "anthropic activities" OR "human activities" OR "anthropogenic activities" OR "anthropic activity" OR "human activity" OR "anthropogenic activity" OR "anthropocene")

Table S1 Each facet of the query was described by a disjunction of keywords separated by "OR" operators. The final query combined all facets using "AND" operators (not shown in the table). Documents were searched starting from 1960. The list of human pressures was adapted from the IPBES Global assessment report (Supplementary materials SM2.1). Field tag DT stands for Document Type, WC for Web of Science Categories, PY for Year Published, and

TS for Topic which includes title, abstract, and keywords. Asterisks (*) represent any group of characters, question marks (?) represent any single character and dollar signs ($) represent zero or one character.

S3. Modified PRISMA flowchart

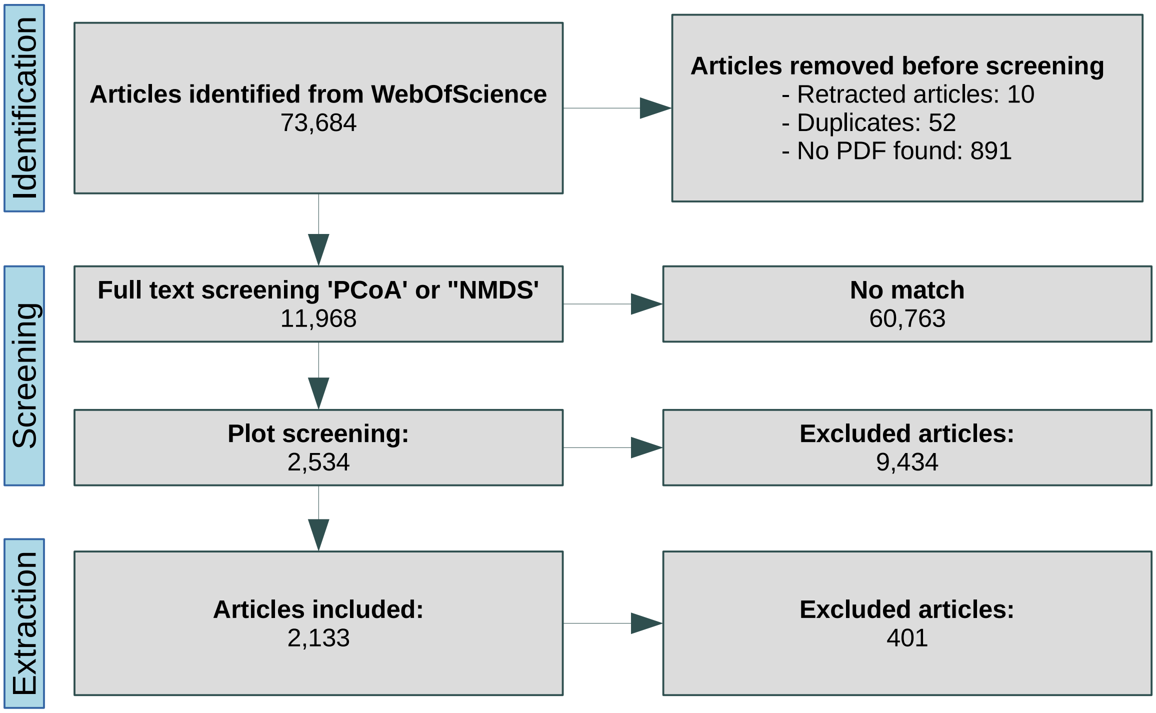

Figure S2 Number of publications included or excluded at each stage of the literature search.

S4. Additional information on data collection and extraction

The metadata of bibliographic references in RIS format and their associated full text in PDF format were centralised and managed in a dedicated Zotero database. The text content of the PDF was extracted using the command-line utility tool pdftotext. The extracted text was analysed to detect any references to PCoA and NMDS analyses by searching for the following regular expression :

```
(?i)NMDS|(?i)PCOA|(?i)principal +coordinate|(?i)multidimensional +scaling
```

The documents containing the target terms were then manually evaluated in order to decide on their inclusion on the basis of a simple rule: presence of at least one biplot graph representing the first two axes of a PCoA or NMDS, and including, while differentiating them, at least two treatments ("control" and "impact"). This procedure was carried out by a single operator. A post-hoc check was carried out on a sub-sample of 100 publications by a second operator. This second operator identified 100% of the publications selected by the main operator (37 in total) while being slightly more conservative by selecting 4 additional references. These references would have been eliminated in any case at the data extraction stage due to the absence of some mandatory information (regarding the contrast of control and impact or other criteria used in the extraction step).

The actual data extraction phase was carried out by the 16 project participants. The extraction methodology was communicated to the participants during two workshops (one face-to-face and one online) and through a briefing document. The workshops included a number of practical demonstrations using a variety of examples to review the various extraction stages and special cases. Face-to-face or remote sessions were then organised, where participants worked together on data extraction and the most complex cases.

The extraction phase was carried out using a dedicated web platform developed specifically for the project. The aim of this platform was to maximise the quality of the user experience while minimising the risk of input errors and offering total control of user privileges to prevent data being exposed. Each participant was provided with a personal account and could add data using an interactive web form. This form supported various features to assist with the data extraction, including required fields, data validation (structures, types, pre-defined options, regular expressions), integration of help in the interface (placeholders and tooltips), and dynamic layout (conditional display of components). The website was built on the content management system Drupal (v.9) and was backed by a MySQL database that connected bibliographic references to data added by users in a seamless and secure way.

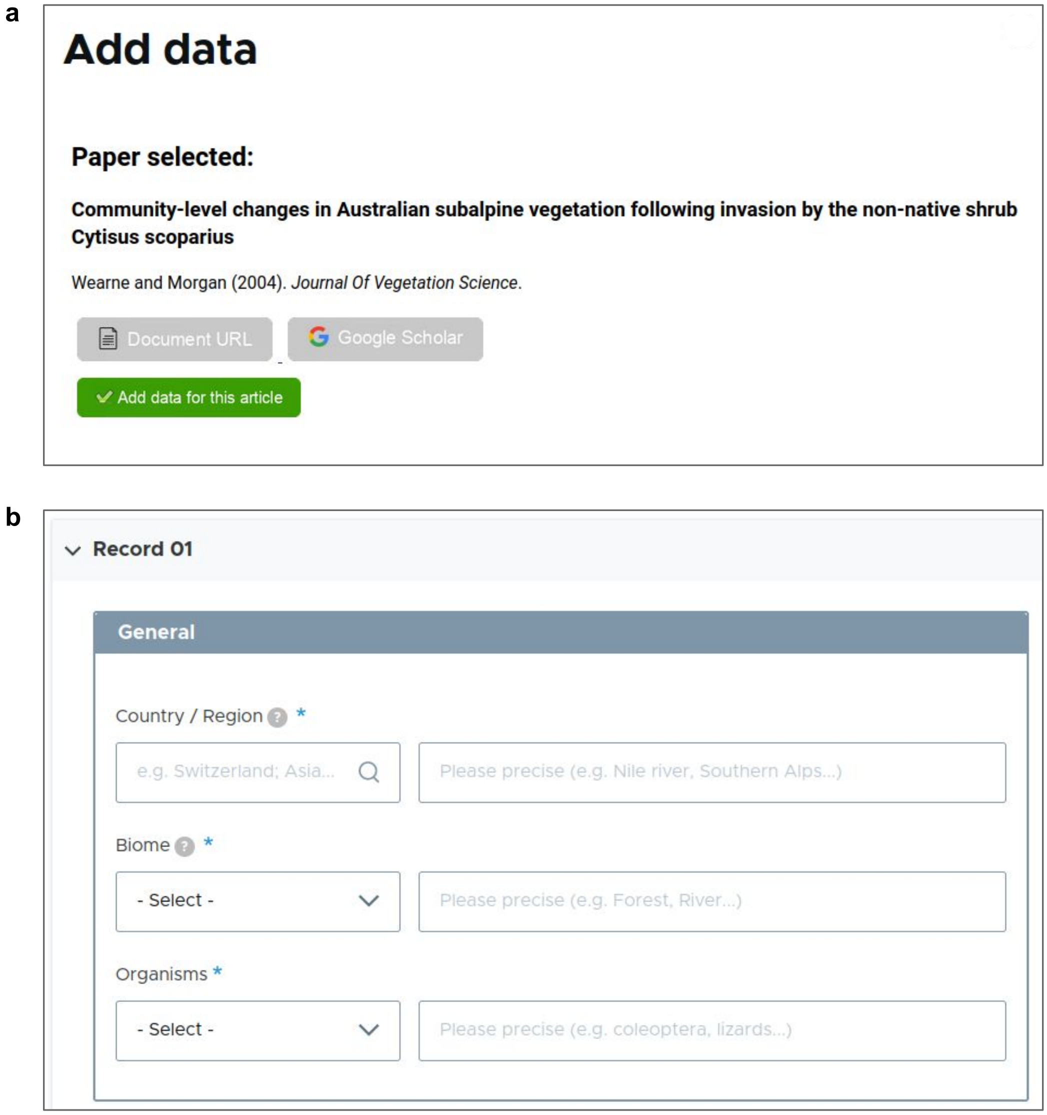

Figure S3 Screenshots of the interface of the web platform developed for data extraction. Connected users were **a**, invited to read and extract data from a publication which was randomly selected from the backend database. If they accepted, they were redirected to **b**, an interactive form designed to assist them with data extraction (only an extract of this form is shown). After they validated the form, data were automatically submitted to the database and users were invited to proceed with a new publication.

S5. Impact of additional samples in PCoA/NMDS on effect size estimation

Our meta-analysis is based on the extraction of sample coordinates from PCoA or NMDS two-dimensional graphs. It is common for several groups of samples or treatments to be represented on the same graph, which led us either to extract several comparisons per graph, or to ignore certain groups of samples (the rules applied for this choice are explained in the "Search strategy" part of the Methods section of the manuscript). The inclusion of additional samples or groups of samples can modify the configuration of the points as calculated by the projection methods, with the possible consequence that the observed distances no longer truly reflect the patterns studied. The impact of other sample groups in the analyses is *a priori* limited since our effect sizes (LRR homogeneity and LRR shift) depend on the relative distances and not the absolute distances between points. We show by simulation that this effect is indeed limited.

Our approach is as follows. We randomly generate a set of communities distributed in n groups ($3 \leq n \leq 5$), each group being defined by its own environmental conditions. We then compute a first PCoA (or NMDS) for the communities belonging to the first two groups, from which we calculate the LRR homogeneity and LRR shift effect sizes. We then compute a second PCoA (or NMDS), this time with all the groups, from which we calculate the LRR homogeneity and LRR shift effect sizes for the first two groups only. The comparison of LRR homogeneity and LRR shift between the two PCoAs allows us to estimate the bias induced by the presence of other groups in the analysis. Details of the community simulation are given in the following pseudo-code (the R code used is available on GitHub).

Listing S1 Algorithm for community simulation

Determine the number of groups, n, an integer randomly chosen in $[3, 4, 5]$.
Determine the number of communities for each group, where each value is an integer randomly chosen in $[20, \dots, 200]$.
Determine the number of environmental dimensions, m, an integer randomly chosen in $[3, 4, 5]$.
Determine the number of species in the pool, s, an integer randomly chosen in $[15, \dots, 100]$, and create S an indexed collection of species of size s.
Create a grouping vector, G, where each element G_i is an integer in $[1, \dots, n]$ and represents the group membership of each communities C_i.
Generate an $s \times m$ random matrix M with each element independently sampled from a uniform distribution in the range $[-1, 1]$, representing the means of the normal distributions of each species along each environmental dimension.

7 Generate an $s \times m$ random matrix V with each element independently sampled from a uniform distribution in the range $[0, 1]$, representing the standard deviation of the normal distributions of each species along each environmental dimension.

8 Generate an $n \times m$ random matrix E containing environmental conditions for each group along each environmental dimension, with each element independently sampled from a normal distribution $\mathcal{N}(\mu = 0, \sigma^2 = 0.05^2)$.

9 For each community C_i belonging to the group G_i, generate species abundances by randomly drawing 300 individuals from S, where for each species S_j the probability to be sampled is weighted by the product of the densities of its normal distributions along each environmental dimension $\forall k \in \{1, \dots, m\}$, $\mathcal{N}(\mu_{j,k} = M_{j,k}, \sigma^2_{j,k} = V^2_{j,k})$ corresponding to the environmental conditions of the community $E_{G_i,k}$.

In total, we simulated 1000 independent sets of communities. Overall, we found a strong correlation between LRR homogeneity derived from the 2 groups analysis and LRR homogeneity derived from the >2 groups analysis (Figure S4a,c). This correlation was $\rho = 0.85$ (t = 51.53, df = 998, P < 0.001) for PCoA and $\rho = 0.70$ (t = 31.176, df = 998, P < 0.001) for NMDS. Figures S5a and S5c show the distribution of the difference between the two variables for the PCoA and NMDS analyses. The mean of this difference is 0.004 for PCoA and -0.007 for NMDS. One sample t-tests show that neither value is statistically different from zero (PCoA: t = 0.984, df = 999, P = 0.325; NMDS: t = -1.371, df = 999, P = 0.170), indicating the absence of bias.

We also found a very strong correlation between the LRR shift values derived from the 2 groups and the >2 groups analyses (Figure S4b,d). This correlation was $\rho = 0.99$ (t = 218.27, df = 998, P < 0.001) for PCoA and $\rho = 0.83$ (t = 31.176, df = 998, P < 0.001) for NMDS. Figures S5b and S9d show the distribution of the difference between the two variables for the PCoA and NMDS analyses. The mean of this difference is -0.035 for PCoA and 0.063 for NMDS. One sample t-tests show that both values are statistically different from zero (PCoA: t = -9.419, df = 999, P < 0.001; NMDS: t = 3.447, df = 999, P < 0.001), indicating a very small but consistent bias, possibly driven in the case of the NMDS by the series of outliers observed in Figures S4d and S5d.

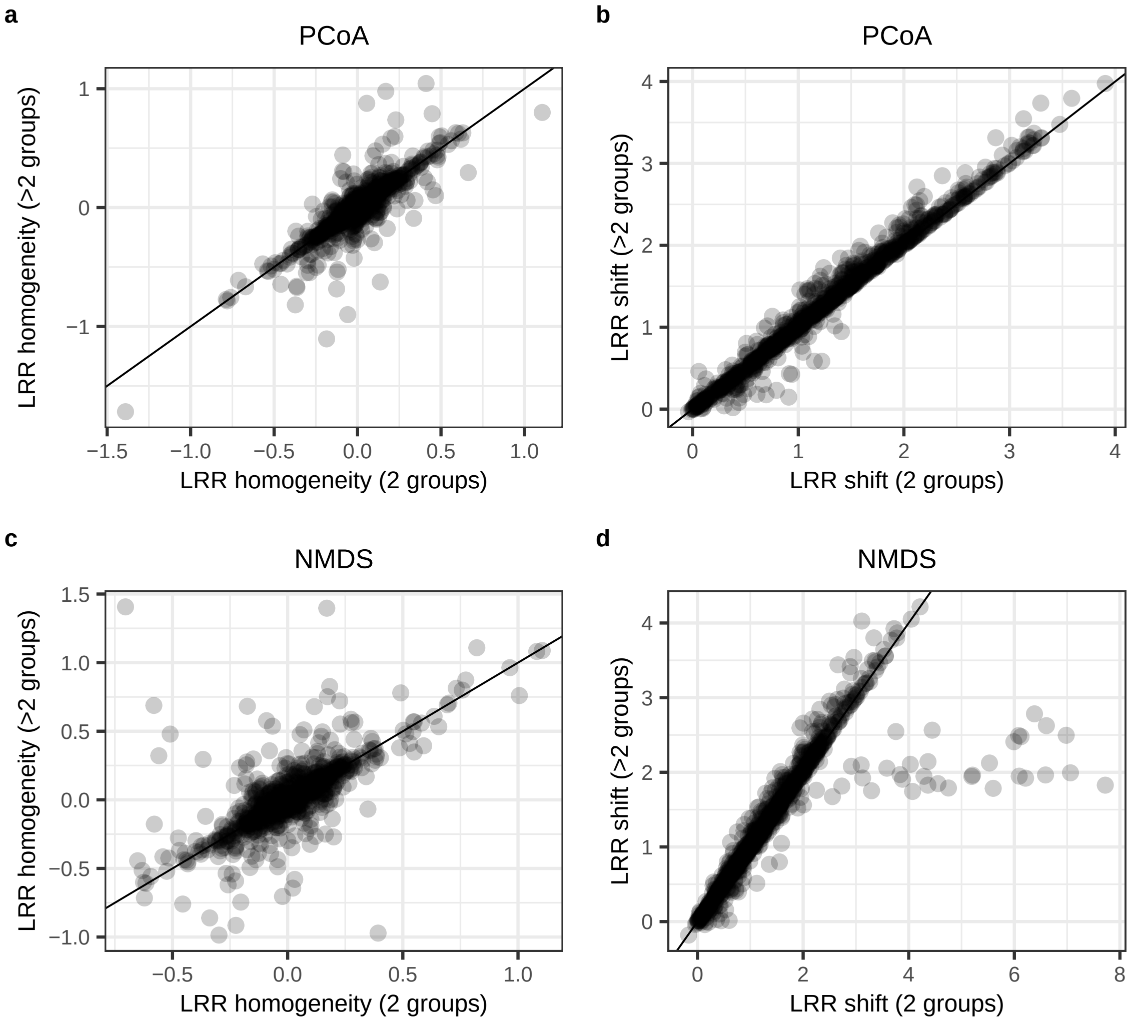

Figure S4 Relationship between the effect-sizes estimated from an analysis based on two groups of points and the same effect-sizes estimated from the same analysis but including additional groups of points. **a**. LRR homogeneity from PCoA, **b**. LRR shift from PCoA, **c**. LRR homogeneity from NMDS and **d**. LRR shift from NMDS. The black line delineates the 1:1 line, corresponding to perfect correspondence between the two estimates.

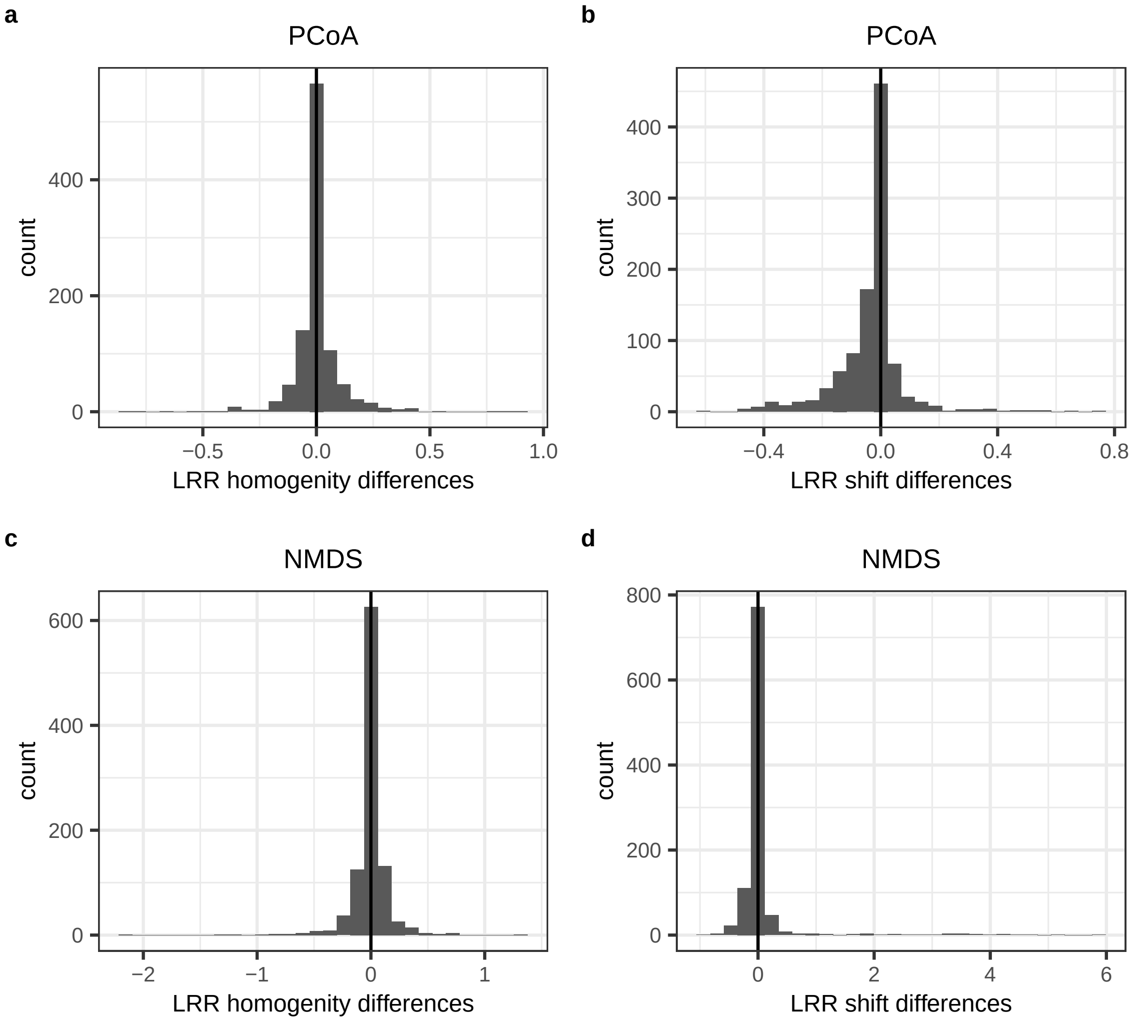

Figure S5 Distributions of the differences between the effect-sizes estimated from an analysis based on two groups of points and the same effect-sizes estimated from the same analysis but including additional groups of points. **a**. LRR homogeneity from PCoA, **b**. LRR shift from PCoA, **c**. LRR homogeneity from NMDS and **d**. LRR shift from NMDS.

S6. Assessment of publication bias

We assessed potential publication bias for each outcome measure, namely LRR homogeneity, LRR shift and LRR local diversity. Our strategy was based on a combination of funnel plot visual inspection[1], file drawer analysis[2] and P-curve analysis[3].

We observe no evidence of asymmetry on the funnel plots with regard to LRR homogeneity (Figure S6a) and LRR local diversity (Figure S6c) while for LRR shift a small asymmetry can be observed (Figure S6b). This asymmetry, however, appears to be linked to the effect size distribution and has no apparent relationship with the standard error. A small-study effect, on the contrary, would have resulted in an over-representation of studies in the lower right-hand part of the funnel plot only.

To ascertain the robustness of our findings against possible publication bias, we performed a File Drawer Analysis and calculated the Fail-Safe N, a measure estimating the number of additional studies with null findings required to render observed effects non-significant. The File Drawer Analysis was conducted with the function `fsn` from the R package `metafor` using the generalised method of Orwin and Rosenberg[2,4]. We found Fail-Safe N values of 19,689 for LRR homogeneity, 2,757,894 for LRR shift and 12,052 for LRR local diversity. Overall these large to very large values (5 to 1000 times more studies than included in our analyses) indicate that our results are robust to publication bias.

Finally, we performed a test for p-values right-skewness (P-Curve analysis) to further validate the robustness of our results, specifically with regards to p-hacking. For every tested effect sizes, we found that both the half and full p-curve tests clearly indicated a right-skewed shape: LRR homogeneity (Full: $Z = -21.8$, $P < 0.001$, Half: $Z = -20.3$, $P < 0.001$), LRR shift (Full: $Z = -62.0$, $P < 0.001$, Half: $Z = -51.5$, $P < 0.001$) and LRR local diversity (Full: $Z = -66.4$, $P < 0.001$, Half: $Z = -67.2$, $P < 0.001$). Together these results indicate that there is strong evidential value behind our data.

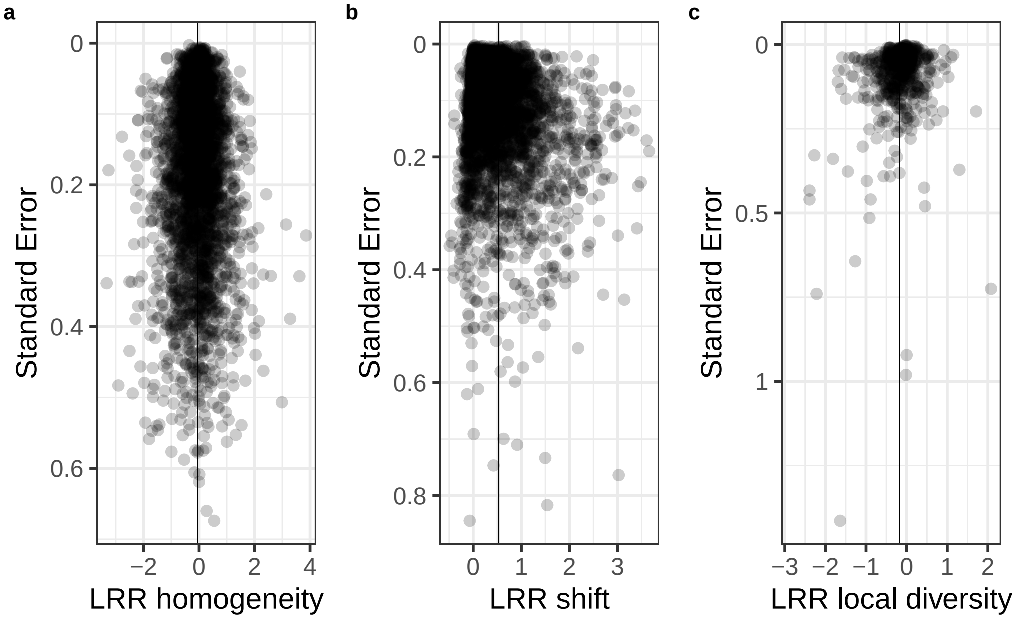

Figure S6 Funnel plots of effect sizes versus standard error for **a**. LRR homogeneity, **b**. LRR shift and **c**. LRR local diversity.

S7. Spatial distribution of reference and impacted sites

We analysed the spatial distribution of reference and impacted study sites in order to compare geographical patterns with those observed in biological communities.

To this end, we extracted the geographical coordinates of reference and impacted sites for a subset of 200 articles. The coordinates of impacted sites were extracted directly from the maps published in the articles, using the same digitising technique described in the manuscript for biological communities. We also used the same effect sizes (LRR homogeneity and LRR shift) here applied to geographic distance matrices to analyse whether 1) distances between samples within treatments were equivalent (LRR homogeneity) and 2) whether distances between treatments were greater than distances within treatments (LRR shift).

Spatial LRR homogeneity exhibited a distribution closely centred around zero (Figure S7a) and a small (0.17) yet significant mean value ($t = 2.979$, $df = 199$, $P = 0.003$). Similarly we found the mean of spatial LRR shift (0.32) significant ($t = 7.087$, $df = 199$, $P < 0.001$) and its distribution right skewed (Figure S7b). However, neither of the two space-based effect-sizes seems clearly correlated with their equivalent community-based measures (LRR homogeneity: $\rho = 0.08$, $t = 1.142$, $df = 198$, $P = 0.25$, Figure S7c; LRR shift: $\rho = 0.13$, $t = 1.888$, $df = 198$, $P = 0.06$, Figure S7d).

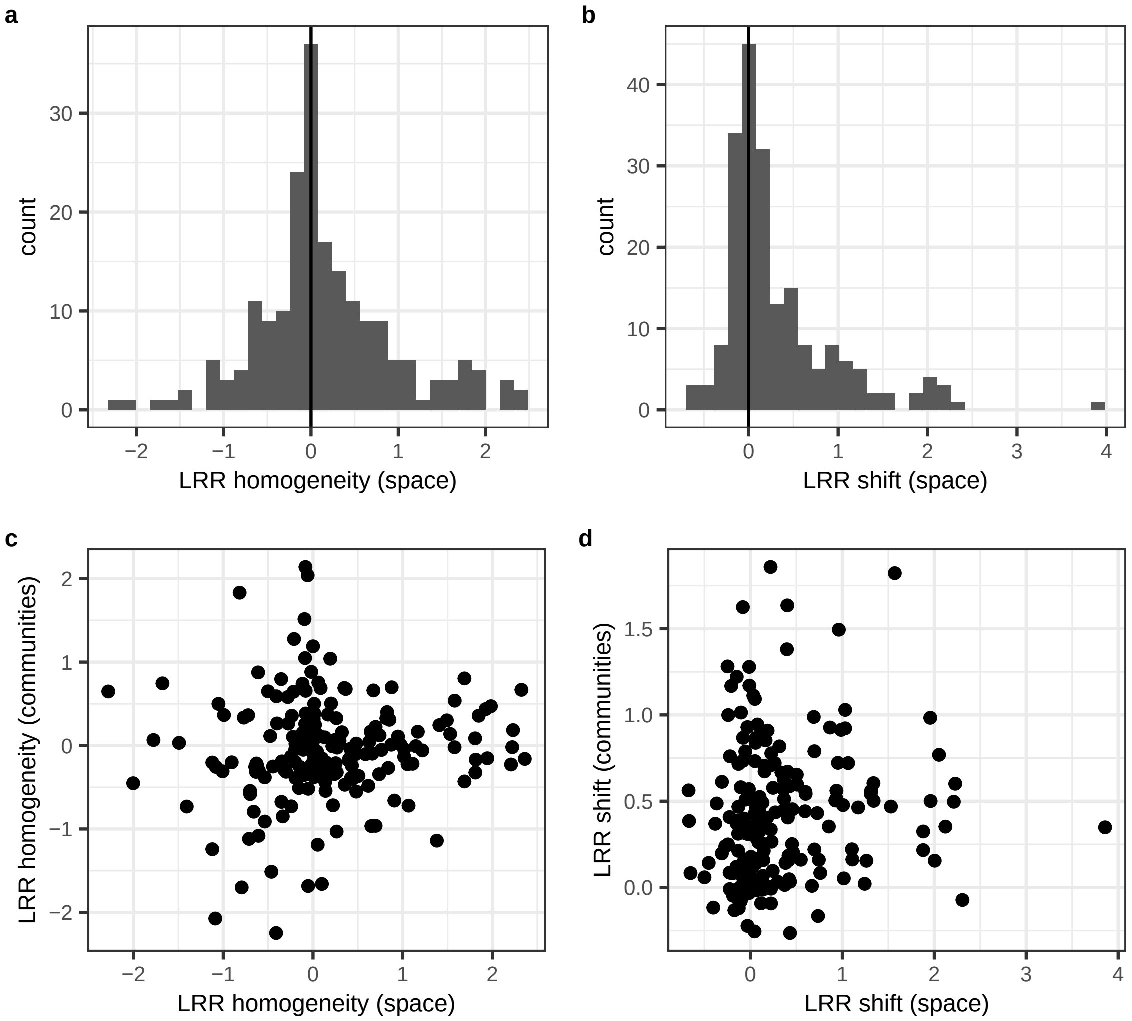

Figure S7 Distributions of **a**. LRR homogeneity and **b**. LRR shift values computed from spatial distances for a subset of 186 studies. Relationship between **c.** LRR homogeneity and **d.** LRR shift values computed from spatial and community distances.

Supplementary References

1. Møller, A. P. & Jennions, M. D. Testing and adjusting for publication bias. *Trends in Ecology & Evolution* **16**, 580–586 (2001).

2. Rosenberg, M. S. The file-drawer problem revisited: A general weighted method for calculating fail-safe numbers in meta-analysis. *Evolution* **59**, 464–468 (2005).

3. Simonsohn, U., Simmons, J. P. & Nelson, L. D. Better p-curves: Making p-curve analysis more robust to errors, fraud, and ambitious p-hacking, a reply to ulrich and miller (2015). *Journal of Experimental Psychology: General* **144**, 1146–1152 (2015).

4. Orwin, R. G. A Fail-SafeN for Effect Size in Meta-Analysis. *Journal of Educational Statistics* **8**, 157–159 (1983).
