# Supplementary material for: The global human impact on biodiversity
Source: Nature. 2025 Mar 26;641(8062):395–400. doi: 10.1038/s41586-025-08752-2 (PMC12058524; doi:10.1038/s41586-025-08752-2)
Supplement: Supplementary file 2 — Reporting Summary [file 41586_2025_8752_MOESM2_ESM.pdf]

nature portfolio

Corresponding author(s): François Keck
Florian Altermatt

Last updated by author(s): Jan 7, 2025

Reporting Summary

Nature Portfolio wishes to improve the reproducibility of the work that we publish. This form provides structure for consistency and transparency in reporting. For further information on Nature Portfolio policies, see our Editorial Policies and the Editorial Policy Checklist.

Statistics

For all statistical analyses, confirm that the following items are present in the figure legend, table legend, main text, or Methods section.

n/a	Confirmed	
☐	☒	The exact sample size (*n*) for each experimental group/condition, given as a discrete number and unit of measurement
☐	☒	A statement on whether measurements were taken from distinct samples or whether the same sample was measured repeatedly
☐	☒	The statistical test(s) used AND whether they are one- or two-sided<br>*Only common tests should be described solely by name; describe more complex techniques in the Methods section.*
☐	☒	A description of all covariates tested
☐	☒	A description of any assumptions or corrections, such as tests of normality and adjustment for multiple comparisons
☐	☒	A full description of the statistical parameters including central tendency (e.g. means) or other basic estimates (e.g. regression coefficient) AND variation (e.g. standard deviation) or associated estimates of uncertainty (e.g. confidence intervals)
☐	☒	For null hypothesis testing, the test statistic (e.g. *F*, *t*, *r*) with confidence intervals, effect sizes, degrees of freedom and *P* value noted<br>*Give P values as exact values whenever suitable.*
☒	☐	For Bayesian analysis, information on the choice of priors and Markov chain Monte Carlo settings
☒	☐	For hierarchical and complex designs, identification of the appropriate level for tests and full reporting of outcomes
☒	☐	Estimates of effect sizes (e.g. Cohen's *d*, Pearson's *r*), indicating how they were calculated

Our web collection on statistics for biologists contains articles on many of the points above.

Software and code

Policy information about availability of computer code

Data collection	Data extraction from the plots was achieved using Webplotdigitizer v. 4.6 in manual mode.
Data analysis	Statistical analyses were conducted with R v.4.0.3. Mixed models were fitted using the glmmTMB package and marginal means estimated using the emmeans package. Code and information about the environment to reproduce the analyses and graphical outputs are available on GitHub (https://github.com/fkeck/metabeta) and are permanently archived on Zenodo (https://doi.org/10.5281/zenodo.14608770).<br>Package version:<br>tidyverse v.2.0.0, readr v.2.1.4, scales v.1.3.0, metafor v.4.4-0, vegan v.2.6-4, patchwork v.1.2.0, MASS v.7.3-55, broom v.1.0.5, car v.3.1-2, broom.mixed v.0.2.9.4, glmmTMB v.1.1.8, emmeans v.1.10.0, ggtext v.0.1.2, jsonlite v.1.8.8, tibble v.3.2.1, grImport2 v.0.3-1, stringr v.1.5.1, glue v.1.7.0, sf v.1.0-15, rphylopic v.1.3.0, ggeffects v.1.5.0, dplyr v.1.1.4, quarto v.1.4

For manuscripts utilizing custom algorithms or software that are central to the research but not yet described in published literature, software must be made available to editors and reviewers. We strongly encourage code deposition in a community repository (e.g. GitHub). See the Nature Portfolio guidelines for submitting code & software for further information.

Data

Policy information about availability of data

All manuscripts must include a data availability statement. This statement should provide the following information, where applicable:

- Accession codes, unique identifiers, or web links for publicly available datasets
- A description of any restrictions on data availability
- For clinical datasets or third party data, please ensure that the statement adheres to our policy

Articles were searched using the Science Citation Index Expanded (SCI-EXPANDED) database accessed through the Web of Science platform (https://www.webofscience.com). Extracted data can be obtained from the GitHub project repository (https://github.com/fkeck/metabeta) permanently archived on Zenodo (https://doi.org/10.5281/zenodo.14608770).

Research involving human participants, their data, or biological material

Policy information about studies with human participants or human data. See also policy information about sex, gender (identity/presentation), and sexual orientation and race, ethnicity and racism.

Reporting on sex and gender	Not applicable.
Reporting on race, ethnicity, or other socially relevant groupings	Not applicable.
Population characteristics	Not applicable.
Recruitment	Not applicable.
Ethics oversight	Not applicable.

Note that full information on the approval of the study protocol must also be provided in the manuscript.

Field-specific reporting

Please select the one below that is the best fit for your research. If you are not sure, read the appropriate sections before making your selection.

☐ Life sciences ☐ Behavioural & social sciences ☒ Ecological, evolutionary & environmental sciences

For a reference copy of the document with all sections, see nature.com/documents/nr-reporting-summary-flat.pdf

Ecological, evolutionary & environmental sciences study design

All studies must disclose on these points even when the disclosure is negative.

Study description	This study is a meta-analysis of studies comparing biodiversity between reference and human impacted sites. The goal of this study is to measure the magnitude of biodiversity changes under anthropogenic pressure and to understand how these changes relate to different types of human activities.
Research sample	All studies reporting results of compositional shift and homogenisation in response to human pressures (systematically comparing impact vs. reference scenarios).
Sampling strategy	Our sample size directly depends on available published material. Our approach aims to be exhaustive (i.e. include all available relevant studies, no subsampling).
Data collection	The data collection was performed by all co-authors using a web platform developed specifically for the project. Data were collected directly from the publications, using the tool Webplotdigitizer v. 4.6 to extract data from images where necessary.
Timing and spatial scale	The initial bibliographic search was performed on the Web of Science database on the 17/01/2022. Data were extracted from publications from June 2022 to February 2023. The publications used in the meta-analysis were published from 1992 to 2022 and cover the entire world (see Fig. 1).
Data exclusions	No data were excluded. Only studies that fulfilled the requirements for the meta-analysis were included (see Methods).
Reproducibility	All data were extracted from published and available material. Extracted data and the code used for the analyses are available on the GitHub repository of the project: https://github.com/fkeck/metabeta.
Randomization	Randomization is not relevant to our study which is a meta-analysis and not an experiment with controlled design.

Blinding: Blinding is not relevant to our study which is a meta-analysis. As such, no external participants were involved.

Did the study involve field work? ☐ Yes ☒ No

Reporting for specific materials, systems and methods

We require information from authors about some types of materials, experimental systems and methods used in many studies. Here, indicate whether each material, system or method listed is relevant to your study. If you are not sure if a list item applies to your research, read the appropriate section before selecting a response.

Materials & experimental systems

n/a	Involved in the study
☒	☐ Antibodies
☒	☐ Eukaryotic cell lines
☒	☐ Palaeontology and archaeology
☒	☐ Animals and other organisms
☒	☐ Clinical data
☒	☐ Dual use research of concern
☒	☐ Plants

Methods

n/a	Involved in the study
☒	☐ ChIP-seq
☒	☐ Flow cytometry
☒	☐ MRI-based neuroimaging

Plants

Seed stocks: Not applicable.

Novel plant genotypes: Not applicable.

Authentication: Not applicable.
